# Microbiological Profile and Clinical Features of Septic Arthritis of the Shoulder: A 10-Year Cohort Single-Centre Study

**DOI:** 10.7759/cureus.51074

**Published:** 2023-12-25

**Authors:** Omer Nasim, Aamir Khalil, Salman Khan, Suraj Kohli, Charalampos Pantelias, Fatima Banoori, Abdullah Durrani, Arsallan Karim, Robert Moverley

**Affiliations:** 1 Trauma and Orthopedics, University Hospitals Dorset NHS Foundation Trust, Poole, GBR; 2 Surgery, Hayatabad Medical Complex Peshawar, Peshawar, PAK; 3 Trauma and Orthopedics, University Hospital Southampton NHS Foundation Trust, Southampton, GBR; 4 General Medicine, Quaid-e-Azam International Hospital, Islamabad, PAK

**Keywords:** orthopedics infection, staphylococcus aureus, microbiological culture, microbiological pattern, periprosthetic joint infection

## Abstract

Introduction

Septic arthritis (SA) constitutes a pressing orthopedic emergency characterized by acute, non-traumatic joint pain. Timely diagnosis and intervention are imperative to avert complications such as chondrolysis and systemic sepsis. The etiology is predominantly hematogenous, necessitating an integrated approach involving surgical and microbiological modalities. Shoulder aspiration and microbiological analysis play pivotal roles in guiding treatment, especially when positive findings prompt more aggressive therapeutic strategies. This study aims to elucidate the nuanced clinical and epidemiological characteristics of septic arthritis in both native and prosthetic joints within a singular institutional cohort over a decade.

Methods

This retrospective case series analysis spanned a 10-year period, focusing on non-prosthetic shoulder joints from January 2012 to July 2021. In this timeframe, only 183 aspirations were performed and sent to the microbiology department for analysis, including cultures, microscopy, and antibiotic sensitivity tests for positive cultures. The study delved into the microbiological profile of infections, encompassing gram stain, culture positivity rates, identification of microorganisms, and antibiotic susceptibility patterns. Additionally, the incidence of primary joint infections with resistant strains, particularly methicillin-resistant *Staphylococcus aureus *(MRSA), was scrutinized. Statistical analysis utilized the SPSS program version 20.0 (IBM Inc., Armonk, New York), with a significance level set at 5%. The project, registered with the trust's clinical audit department (Reg #5372), adhered to the Declaration of Helsinki and good clinical practice guidelines. Data collection involved extracting non-identifiable patient modifiers from the laboratory database bank into Excel spreadsheets.

Results

The study included 183 patients, with 108 (59%) females and 75 (41%) males. The average age was 76.2±16.5 years. Among them, 138 (75.4%) reported pain, and 15 (8.2%) had a body temperature over 37.8°C. Lab results showed a mean white blood cell count of 11.6±4.5 and an average C-reactive protein level of 121.7±102.1. Leucocytosis (>11,000 WBC) was seen in 82 (44.8%) cases. Elevated C-reactive protein (CRP; >10 mg/dl) was found in 136 (74.3%) patients. Synovial fluid analysis revealed no crystals in 91.3% of cases. Microbial resistance analysis showed 19 strains resistant to co-trimoxazole and 11 to erythromycin. Among co-trimoxazole-resistant strains, 73.7% were *Staphylococcus aureus*, a statistically significant association (p<0.001).

Conclusion

The evolving sensitivity patterns of microbes in septic arthritis underscore the necessity to reassess empirical antibiotic therapy. Subsequent joint damage resulting from infection can result in substantial disability.

## Introduction

Septic arthritis is more common in children, the elderly, and males [1]. The estimated incidence in industrialized countries is 2-6 cases per 100,000 person-years, varying by region, socioeconomic status, and age [2,3]. The mortality rate in hospitalized patients is 2-10% [4-5]. Acute septic arthritis usually presents with a one-to-two-week history of malaise, erythema, swelling, tenderness, and decreased range of motion in a single joint [6], but these symptoms may be absent [7]. Fever is common; 30-40% have a temperature greater than 39°C [8]. Septic arthritis is mostly monoarticular, but polyarticular disease (10-20% of cases) should be considered, especially in afebrile patients or those with rheumatoid arthritis (RA) [9]. The diagnosis of septic arthritis relies on clinical symptoms, history, examination, and investigations [10]. A careful examination by an experienced clinician is crucial for a rapid diagnosis [11]. Blood tests show increased erythrocyte sedimentation rate (ESR), C-reactive protein (CRP), and WBC. However, normal acute-phase reactants do not rule out septic arthritis [12,13]. Septic arthritis mortality varies (approx. 11% for monoarticular arthritis) [14]. Permanent joint function loss risk is nearly 40% [15]. Delayed diagnosis, advanced age, joint diseases, and synthetic material possess poor prognosis. A delay in management, as little as one week, leads to poor outcomes [16]. Underlying joint diseases have a poor prognosis due to delayed diagnosis [9]. The primary treatment involves prompt debridement and early antibiotic therapy [14].

The incidence may have increased due to orthopedic procedures, aging, and immunosuppression [17]. Except for children under two years, *S. aureus* is the most frequent organism (37-56%) [18,19]. Methicillin-resistant *Staphylococcus aureus *(MRSA) infections have increased in some populations and regions, with newer resistant strains emerging [5,20,21]. *Streptococcus *spp. are the second most common organisms in adults [22,2,20]. *S. pyogenes* is often associated with autoimmune, skin, and trauma conditions [17,13,23]. Group B streptococci are common in elderly patients with chronic diseases such as diabetes [24]. Gram-negative cocci cause about 20% of septic arthritis cases, mainly *N. gonorrhoeae* and *N. meningitidis* [16]. Gram-negative bacilli infections cause 10-20% of septic arthritis cases. They usually affect the young, the old, intravenous drug users, and immunocompromised patients [25]. In patients with diabetes and prostheses or trauma, anaerobic microbes are isolated in a few cases [26]. In HIV-infected patients, *S. aureus* is the most common pathogen, followed by opportunistic pathogens (30%) [27].

Pathogens enter the synovium through hematogenous, contiguous, soft tissue, or direct routes. The well-vascularised nature and lack of a basement membrane facilitate bacterial access [28]. Bacteria multiply in the synovial fluid (SF) and trigger acute inflammation. The host releases inflammatory cytokines, such as IL-1b and IL-6, that promote opsonization and complement activation [29]. In immunocompetent hosts, inflammation clears infection. Otherwise, high cytokine levels may cause joint damage. There is joint effusion, which increases intra-articular pressure, leading to synovium and cartilage destruction.

Risk factors for developing septic arthritis include extremes of ages, i.e., elderly (>80 years) and very young patients [2], and underlying joint diseases, such as rheumatoid arthritis, osteoarthritis, crystal arthropathies, and other inflammatory arthritides [2]. Rheumatoid arthritic patients have a 10-fold higher incidence than the general population [2,17]. Immunosuppressive therapy, glucocorticoids, classic disease-modifying antirheumatic drugs (DMARDs), and anti-tumor necrosis factor (TNF) agents are also risk factors [30,31]. Chronic and immunosuppressive diseases, such as diabetes, leukemia, cirrhosis, cancer, and hypogammaglobulinemia, increase the risk of septic arthritis [7]. Skin infections [2] and HIV-infected patients [32] have a higher prevalence of musculoskeletal infections. Septic arthritis due to intra-articular injection is uncommon with an estimated prevalence of four cases per 10,000 injections [9].

Gram staining of synovial fluid helps diagnose septic arthritis and differentiate gram-positive and gram-negative bacteria for antibiotic therapy. For definitive diagnosis, synovial fluid is cultured for aerobic and anaerobic bacteria, mycobacteria, and fungi. Gram staining is positive in only 50% of cases, while SF cultures are positive in 67% of non-gonococcal arthritis cases [18]. Polarizing light microscopy for negatively birefringent (uric acid) and positively birefringent (calcium pyrophosphate dihydrate) crystals to rule out crystalline joint disease, which does not exclude coexisting septic arthritis [33]. Blood cultures are positive in 50-70% of patients with non-gonococcal arthritis if blood is taken before starting antibiotics [18]. 

Plain radiographs may appear normal initially; however, osteopenia and joint space narrowing are radiological manifestations in later stages. In terms of radiological imaging, CT may not show abnormalities in early infection, but it better visualizes edema, erosions, osteitis, and sclerosis [34]. MRI provides better resolution for joint effusion and bone and soft-tissue infection [35]. MRI findings in septic arthritis include effusion, destruction, abscesses, edema, and cortical interruption. MRI cannot differentiate infective and other inflammatory arthritides [36].

There is a lack of evidence on antibiotic choice and duration due to the limited randomized controlled trials. Early antibiotic treatment should be guided by clinical presentation, suspected organisms, and gram-staining results [8,11]. Considering the prevalence of *S. aureus* and streptococci, initial antibiotics should effectively target these pathogens [8]. The initial antibiotic regimen should be adjusted based on culture and sensitivity results. Apart from antibiotics, septic arthritis management includes the timely removal of purulent material from the joint space, preferably through needle aspiration [37]. Successful outcomes have been observed within the first week of treatment [37,38].

Arthroscopic or open drainage is advised for patients with Incomplete needle aspiration and persistent effusion beyond seven days. Arthroscopy is a less invasive option for accessing deep joints [39]. During the acute infection phase, optimal joint positioning is crucial to prevent subsequent deformities and contractures. Splints can help maintain the joint's correct position, and exercises are necessary to prevent muscular atrophy. After the acute phase, early physical therapy and mobilization of the affected joint are imperative for optimal recovery [8,40].

## Materials and methods

This study was designed as a retrospective case series analysis. Patients were treated at Poole Hospital, Poole, United Kingdom, which accepts acute orthopedic admissions. Data was collected from January 2012 to July 2021. All patients with suspected primary shoulder joint septic arthritis were aspirated during this period. The septic arthritis diagnosis adhered to Newman's criteria [10], requiring the presence of at least one positive criterion: (i) isolation of a pathogenic organism from the affected joint; (ii) isolation of a pathogenic organism from another source (e.g., blood) in the context of a warm, red joint suggestive of sepsis; (iii) typical clinical features and cloudy joint fluid in the presence of prior antibiotic treatment; and (iv) post-mortem or pathological features indicative of septic arthritis. Exclusion criteria comprised individuals below 18 years old, those with a history of joint surgery (including knee replacements), symptoms persisting for over six months, and a diagnosis of tuberculous or fungal arthritis.

The demographic profile of the patients and relevant clinical data were gathered from inpatient medical records, such as the duration of local symptoms (joint pain, swelling) and the presence of fever, comorbidities, immunosuppression, previous joint disease, systemic and orthopedic complications, and length of hospital stay. Laboratory parameters analyzed included C‑reactive protein (CRP) and total and differential white blood cell (WBC) counts in blood.

The microbial characteristics of the infections were scrutinized, encompassing factors such as gram stain results, rates of culture positivity, identification of organisms in cultures, and patterns of antibiotic susceptibility. Additionally, an analysis was conducted on the occurrence of primary joint infections caused by resistant strains, with a specific focus on MRSA.

Statistical analysis

Continuous variables were displayed by providing their respective means and standard deviations. Categorical variables were represented using absolute values and their corresponding percentages. To evaluate the statistical correlation between different categorical variables, the chi-squared test was utilized, and then univariate analysis was done. For continuous variables, the appropriate statistical test was chosen based on the data distribution. Specifically, the non-paired Student's t-test was applied when the data exhibited a parametric distribution, and the Mann-Whitney test was used for non-parametric distributions. The statistical analysis was carried out using the SPSS program version 23.0 (IBM Inc., Armonk, New York), with a significance level of 5% considered for all tests.

Ethical statement

The data collected formed the laboratory database, with extraction in an Excel spreadsheet (Microsoft, Redmond, Washington). There was no additional patient contact, and as such, this project was performed as a service evaluation without the need for formal ethical approval. The project was registered with the trust's clinical audit department (Reg #5372) and was conducted per the Declaration of Helsinki and the guidelines for good clinical practice.

## Results

The study encompassed a cohort of 183 patients, with gender distribution indicating 108 (59%) females and 75 (41%) males. The average age of the patients was 76.2±16.5 years (Table 1). Among the patients, 138 (75.4%) reported experiencing pain, while a subset of 15 (8.2%) exhibited elevated body temperatures exceeding 37.8°C. In terms of laboratory findings, the mean white blood cell count (WBC) stood at 11.6±4.5, and the average C-reactive protein (CRP) level was 121.7±102.1. Leucocytosis, defined as a leukocyte count surpassing 11,000, was evident in 82 (44.8%) cases. Furthermore, 136 (74.3%) patients displayed markedly elevated CRP levels, exceeding 10 mg/dl.

**Table 1:** Epidemiological and clinical characteristics of the joint aspirations CRP - C-reactive protein; WBC - white blood cell

Gender	N (%)
Male	75 (41%)
Female	108 (59%)
Age	76.2±16.5
Clinical features	
Pain	138 (75.4%)
Fever	15 (8.2%)
Laboratory levels	
Mean WBC	11.6±4.5
Leucocytosis	82 (44.8%)
CRP	121.7±102.1
Marked elevated CRP	136 (74.3%)
Metalwork	19 (10.4%)
Crystals on microscopy	
No crystals seen	167 (91.3%)
Sodium urate	3 (1.6%)
Calcium pyrophosphate	9 (4.9%)
Previous joint disease	
Rheumatoid arthritis	24(13.1%)
Osteoarthrosis	58 (31.7%)
Psoriatic arthritis	3 (1.6%)
Immune status	
Use of steroids or immunosuppressants	42 (23%)
Chronic kidney disease	11 (6.0%)
Neoplasms	24 (13.1%)
Hepatic cirrhosis	1 (0.5%)
Comorbidities	
Systemic hypertension	91 (49.7%)
Diabetes	23 (12.6%)
Systemic diseases	13 (7.1%)
Clinical complication	
Death	8 (4.4%)
Septic shock	2 (1.1%)
Acute myocardial infarction	2 (1.1%)
Pulmonary complication	1 (0.5%)
Orthopedic complication	
Osteoarthrosis	32 (17.5%)
Rigidity	19 (10.4%)
Chronic osteomyelitis	4 (2.2%)
Surgical wound complication	5 (2.7%)
Rotator cuff tear	23 (12.6%)
Osteochondral lesion	3 (1.6%)
Length of hospital stay (mean±SD)	10.9±15.9

Among the patients, 85 individuals (46.4%) had a history of previous joint ailments. Further examination within this subgroup unveiled specific diagnoses, with 24 patients (13.1%) identified as having rheumatoid arthritis, 58 patients (31.7%) diagnosed with osteoarthrosis, and three patients (1.6%) presenting with psoriatic arthritis. Moreover, comorbid conditions were evident in 127 (69.3%) patients, with 91 (49.7%) of them having hypertension and 23 (12.6%) being diabetic. Systemic illnesses were detected in 13 (7.1%) cases. Within the patient pool, 78 (42.6%) individuals were immunocompromised, primarily attributed to the use of corticosteroids or other immunosuppressive agents in 42 (23%) cases. Chronic renal failure was present in eleven of the immunocompromised patients. Notably, clinical complications were observed in 13 (7.1%) patients, encompassing events such as eight deaths, two instances of septic shock, two cases of acute myocardial infarction, and one pulmonary complication. Orthopedic complications were noted in 86 (46.9%) patients, which included rigidity in 19 patients, osteoarthrosis in 32 patients, chronic osteomyelitis in four patients, surgical wound complications in five patients, rotator cuff tears in 23 patients, and osteochondral lesions in three patients (Table 1).

Microscopic examination of synovial fluid samples to identify crystals revealed specific findings: the absence of crystals was noted in the majority, with 91.3% of cases showing no crystal presence. Sodium urate crystals were detected in a minor percentage, representing 1.6% of cases, while calcium pyrophosphate crystals were identified in 4.9% of cases. Gram staining of the synovial fluid indicated the presence of bacteria in a limited subset, accounting for only 18 (9.8%) cases. However, microbial cultures provided positive results for a higher number of patients, totaling 49 (26.7%). It is noteworthy that a disparity between the results of gram staining and culture was evident in 31 patients. *Staphylococcus aureus* emerged as the most frequently isolated organism, identified in 21 (11.5%) patients. Group A streptococcus was found in two (1.6%) cases, while* Staphylococcus epidermidis* was noted in five (2.7%) patients. Mixed coagulase-negative staphylococci were observed in six (3.3%) cases, *Escherichia coli (E. coli)* was detected in two (1.1%) patients and other streptococcus species were identified in 13 (7.1%) patients, as detailed in Table 2.

**Table 2:** Microbiological characteristics of the joint aspirations MRSA - methicillin-resistant *Staphylococcus aureus*

Gram stain	N (%)		
Gram-positive	18 (9.8%)		
No organism seen	160 (87.4%)		
Organism isolated (%)			
Staphylococcus aureus	21 (11.5%)		
Group A streptococcus	2 (1.6%)		
Staphylococcus epidermidis	5 (2.7%)		
Mixed coagulase negative	6 (3.3%)		
Escherichia coli	2 (1.1%)		
Other streptococcus	13 (7.1%)		
Antibiotic sensitivity	Sensitive (n)	Resistant (n)	Intermediate (n)
Flucloxacillin	8	-	-
Erythromycin	32	11	1
Clindamycin	23	3	4
Doxycycline			-
Ciprofloxacin	7	5	-
Co-trimoxazole	18	19	-
Penicillin	8	-	-
Oxacillin	5	1	-
Ceftazidime	23	3	-
Meropenem	4	1	-
Piperacillin-tazobactam	2	-	-
Ampicillin	2	1	-
Cefuroxime	10	-	-
Co-amoxiclav	28	6	1
Tetracycline	6	-	-
Moxifloxacin	29	-	-
Clarithromycin	27	5	-
Rifampicin	27	2	-
Gentamycin	22	7	-

Analysis of microbial antibiotic resistance unveiled that out of the tested strains, 19 exhibited resistance to co-trimoxazole, and 11 demonstrated resistance to erythromycin. Among the strains resistant to co-trimoxazole, a significant majority, specifically 14 out of 19 (73.7%), were identified as *Staphylococcus aureus*. This observed association was statistically significant (p<0.001).

The univariate statistical analysis uncovered noteworthy findings regarding the occurrence of orthopedic and clinical complications among patients with specific characteristics. Patients who had osteoarthrosis as a previous joint disease and those with diabetes exhibited a notably higher incidence of orthopedic complications, with a statistical significance level of p<0.01, as depicted in Table 3. Moreover, patients who experienced clinical complications had a significantly prolonged average hospitalization duration, amounting to 29±31.4 days, in contrast to the shorter stay of 9.5±13.2 days for those without complications (p<0.01). Furthermore, the presence of at least one comorbidity, particularly neoplasm, exhibited a significant association with the occurrence of clinical complications (p<0.01), as outlined in Table 3.

**Table 3:** Patients prognostic factors for clinical and orthopaedic complications CRP - c-reactive protein; AIDS - acquired immune deficiency syndrome; MRSA - methicillin-resistant *Staphylococcus aureus*

Parameters	Clinical complication		p-value	Orthopedic complication		p-value
	yes	No		Yes	No	
Age, mean±SD	80.4±16.5	75.8±16.5	0.3	75.76±17.6	76.61±15.66	0.7
Gender, N (%)						
Male	8 (10.7%)	67 (89.3%)	0.1	33 (44%)	42 (56%)	0.3
Female	5 (4.6%)	103 (95.4%)		53 (49.1%)	55 (50.9%)	
Clinical features						
Pain	8 (5.8%)	130 (94.2%)	0.07	71 (51.4%)	76 (48.6%)	0.1
Fever	3 (20%)	12 (80%)		5 (33.3%)	10 (66.7%)	
Laboratory levels						
Leucocytosis	6 (7.3%)	76 (92.7%)	0.56	38 (46.3%)	44 (53.7%)	0.5
WBC (mean±SD)	13.86±6.7	11.5±4.3	0.1	11.9±5.1	11.4±4	0.08
Marked elevated CRP	12 (8.8%)	124 (91.2%)	0.3	65 (47.8%)	71 (52.2%)	0.08
CRP (mean±SD)	279.2±126.6	107±87.6	<0.01	106.1±79.8	134.86±116.5	0.42
Crystals on Microscopy						
No crystals seen	11 (6.6%)	156 (93.4%)	0.18	78 (46.7%)	89 (53.3%)	0.18
Sodium urate	0 (%)	3 (100%)		3 (100%)	0 (%)	
Calcium pyrophosphate	2 (22.2%)	7 (77.8%)		4 (44.4%)	5 (55.6%)	
Previous joint disease						
Rheumatoid arthritis	0 (%)	24 (100%)	0.1	11 (45.8%)	13 (54.2%)	0.5
Osteoarthrosis	5 (8.6%)	53 (91.4%)	0.3	39 (67.2%)	19 (32.8%)	<0.01
Psoriatic arthritis	0 (%)	3 (100%)	0.8	1 (33.3%)	2 (66.7%)	0.5
Immune status						
Use of steroids or immunosuppressants	0 (%)	42 (100%)	0.1	20 (47.6%)	22 (52.4%)	0.5
Chronic kidney disease	2 (18.2%)	9 (81.8%)	0.1	4 (36.4%)	7 (63.3%)	0.3
Neoplasms	5 (20.8%)	19 (79.2%)	0.01	11 (45.8%)	13 (54.2%)	0.5
Hepatic cirrhosis	0 (%)	1 (100%)	0.9	0 (%)	1 (100%)	0.5
Comorbidities						
Systemic hypertension	8 (8.8%)	83 (91.2%)	0.2	35 (38.5%)	56 (61.5%)	0.08
Diabetes	3 (13%)	20 (87%)	0.2	14 (60.9%)	9 (39.1%)	<0.01
Systemic diseases	0 (%)	2 (100%)	0.8	0	0	
Other	0 (%)	11 (100%)	0.4	10 (90.9%)	1 (9.1%)	<0.01
Clinical complication						
Death	-	-		0	8 (100%)	0.7
Septic shock	-	-		1 (50%)	1 (50%)	0.7
Acute myocardial infarction	-	-		1 (50%)	1 (50%)	0.7
Pulmonary complication	-	-		0	1 (100%)	0.5
Acute kidney injury	-	-		0	0	
Orthopedic complication						
Osteoarthrosis	2 (6.3%)	30 (93.8%)	0.5	-	-	
Rigidity	0 (%)	19 (100%)	0.2	-	-	
Chronic osteomyelitis	0 (%)	4 (100%)	0.7	-	-	
Rotator cuff tear	0 (%)	23 (100%)	0.1			
Surgical wound complication	0 (%)	5 (100%)	0.6	-	-	
Days in hospital (mean±SD)	29±31.4	9.5±13.2	<0.01	10.8±17.3	11±14.6	0.94
Bacteria isolated						
Staphylococcus aureus	3 (14.3%)	18 (85.7%)	0.1	9 (42.9%)	12 (57.1%)	0.4
Group B streptococcus	2 (66.7%)	1 (33.3%)	0.01	0	3 (100%)	0.1
Other Streptococcus	1 (6.7%)	14 (93.3%)	0.7	8 (53.3%)	7 (46.7%)	0.4
Staphylococcus epidermidis	0 (%)	5 (100%)	0.6	2 (40%)	3 (60%)	0.5
Mixed coagulase negative	1 (16.7%)	5 (83.3%)	0.3	1 (16.7%)	5 (83.3%)	0.1
Escherichia coli	1 (50%)	1 (50%)	0.1	1 (50%)	1 (50%)	0.7

Univariate statistical analysis unveiled significant associations between several factors and the presence of bacteria in patients' synovial fluid cultures. Specifically, the use of steroids exhibited a significant correlation with bacterial culture positivity (p=0.02). Additionally, patients diagnosed with rheumatoid arthritis (p=0.04) and chronic osteomyelitis (p=0.01) were found to be correlated with bacterial isolation. It is noteworthy that patients with positive bacterial cultures experienced a notably extended mean hospitalization period of 14.9±13.3 days in comparison to 9.3±16.6 days for those without positive cultures, and this difference was statistically significant (p<0.01). Furthermore, the presence of bacterial growth was associated with elevated mortality rates among patients (p=0.01), as outlined in Table 4.

**Table 4:** Bacteria isolated vs clinical features of the joint aspiration

Parameters	Bacteria isolated		p-value
	Yes	No	
Age (mean±SD)	71.3±18.2	78.1±15.5	0.01
Gender N (%)			
Male	31 (41.3%)	44 (58.7%)	<0.01
Female	21 (19.4%)	87 (80.6%)	
Clinical features			
Pain	42 (30.4%)	96 (69.6%)	0.5
Fever	4 (26.7%)	11 (73.3%)	
Laboratory levels			
Leucocytosis	30 (36.6%)	52 (63.4%)	0.08
Marked elevated CRP	46 (33.8%)	90 (66.2%)	0.1
Crystals on microscopy			
No crystals seen	48 (28.7%)	119 (71.3%)	0.1
Sodium urate	1 (33.3%)	2 (66.7%)	
Calcium pyrophosphate	0 (%)	9 (100%)	
Previous joint disease			
Rheumatoid arthritis	3 (12.5%)	21 (87.5%)	0.04
Osteoarthrosis	15 (25.9%)	43 (74.1%)	0.3
Psoriatic arthritis	1 (33.3%)	2 (66.6%)	0.63
Immune status			
Use of steroids or immunosuppressants	6 (14.3%)	36 (85.7%)	0.01
Chronic kidney disease	4 (36.4%)	7 (63.6%)	0.38
Neoplasms	10 (41.7%)	14 (58.3%)	0.09
Hepatic cirrhosis	0 (%)	1 (100%)	0.7
Comorbidities			
Systemic hypertension	21 (23.1%)	70 (76.9%)	0.07
Diabetes	10 (43.5%)	13 (56.5%)	0.07
Systemic diseases			
Other	4 (36.4%)	7 (63.6%)	0.3
Clinical complication			
Death	5 (62.5%)	3 (37.5%)	0.04
Septic shock	1 (50%)	1 (50%)	0.4
Acute myocardial infarction	1 (50%)	1 (50%)	0.4
Pulmonary complication	1 (100%)	0 (%)	0.2
Acute kidney injury	0 (%)	0 (%)	
Orthopedic complication			
Osteoarthrosis	8 (25%)	24 (75%)	0.4
Chronic osteomyelitis	2 (50%)	2 (50%)	0.01
Rigidity	1 (5.3%)	18 (94.7%)	0.3
Surgical wound complication	2 (40%)	3 (60%)	0.4
Days in hospital (mean+-SD)	14.9±13.3	9.3±16.6	0.03
Gram stain			
Gram-positive	18 (100%)	0 (%)	<0.01
No organism seen	30 (18.8%)	130 (81.3%)	

## Discussion

This research study offers a clinical and epidemiological assessment of 183 cases, featuring a mean age of 76.2±16.5 years. This age distribution is consistent with earlier investigations [41-43] that have reported an average age exceeding 60 years. Within the studied population, patients were found to be immunosuppressed, had a history of previous joint disease, and or exhibited a comorbid condition. Previous research has established an association between septic arthritis and compromised immune systems [41-43], comorbidities [42-46], as well as previous joint diseases such as osteoarthrosis and rheumatoid arthritis, and this study has had a similar presentation [22].

Within the scope of this investigation, it was noted that around one-tenth of individuals had a diagnosis of rheumatoid arthritis (RA), which was nearly double the prevalence compared to prior studies reporting an occurrence of RA among patients with septic arthritis. However, it is essential to acknowledge that the previous studies encompassed cases involving the elbow and shoulder joints in conjunction, thus, cases might have had a bias [47]. Moreover, the cases involved in this cohort of patients with rheumatoid arthritis, the usage of steroids and immunosuppressants, and the presence of diabetes all historically being risk factors for bacterial isolation in culture in joints. Rheumatoid arthritis has been identified as a notable risk factor for septic arthritis, estimating a four- to eight-fold increase in its incidence among patients with RA in similar European populations [12,48,49].

Additionally, a significant increase in C-reactive protein (CRP) levels, exceeding 10mg/dL, was noted in two-thirds of the patients, a result that closely aligns with the findings of another study conducted [48] where elevated CRP levels were observed in greater than ninety percent of the participants. Leucocytosis, defined by an elevated white blood cell count, was detected in approximately half of the cases, slightly surpassing other studies [44] and comparable to other authors [44,22]. The mean C-reactive protein (CRP) in our study was raised slightly, exceeding the findings of other studies [50], but the CRP that was considered in this study was taken at the first presentation before the joint aspirate collection for analysis.

There exists a prevailing reliance on gram staining, with studies indicating a frequency of positively identifying the etiologic agent more than twice that observed in this cohort, constituting an intriguing observation [51]. Regarding synovial fluid microscopy, our investigation disclosed that approximately one percent of patients manifested sodium urate crystals, while calcium pyrophosphate crystals were observed in nearly five percent. In contrast, extant literature posits a higher prevalence, approximately 10 percent for sodium urate crystals and five percent for calcium pyrophosphate crystals in septic arthritis patients [50]. Notably, in our study, the etiologic agent was identified through synovial fluid culture in less than thirty cases, whereas other investigations have reported identification rates ranging from eighty to ninety-five percent for the responsible bacteria [22,41,42,48,52]. This underscores the evolving nature of trends, necessitating ongoing assessment of previous patterns to enhance comprehension of continually changing microbiological profiles.

The predominant pathogen in this series was *Staphylococcus aureus*, detected in 11.5% of patients with positive synovial fluid cultures. This microbial profile is consistent with other articles where S. aureus was responsible for 42% to 77% of infections [53-55]. In terms of microbial antibiotic resistance, the analysis in this study revealed that among the tested strains, 19 exhibited resistance to co-trimoxazole, and 11 were resistant to erythromycin. Notably, the majority (73.7%) of co-trimoxazole-resistant strains were identified as *Staphylococcus aureus*.

Within this study, 46.9% of patients experienced orthopedic complications during the follow-up period, primarily characterized by osteoarthrosis (17.5%) and rotator cuff tear (12.6%). These rates closely mirror findings from a previous study [47], where 15% of patients developed osteoarthrosis and 9% experienced rotator cuff tears following treatment for shoulder septic arthritis. Notably, patients with a history of rheumatoid arthritis or diabetes exhibited a significantly elevated occurrence of orthopedic complications. While the available literature did not yield specific data on age-related or comorbidity-related variations in the incidence of orthopedic complications in septic arthritis, certain studies have identified risk factors associated with age and comorbidities. For example, septic arthritis is more prevalent among immunosuppressed individuals, those using immunosuppressants, and those with comorbidities like diabetes [15]. In the context of this study, 7.1% of patients experienced clinical complications during their hospitalization, with the most frequent being mortality, observed in 4.4% of patients. Other complications included acute myocardial infarction (AMI) and septic shock, each occurring in 1.1% of patients. Patients who developed clinical complications had hospitalization durations approximately three times longer than those without complications. The observed mortality rate of 4.4% during hospitalization was slightly lower than rates reported in studies involving septic arthritis affecting multiple joints, where mortality ranged from 6% to 11.5% [38,22]. However, these results closely resembled findings in studies specifically focused on shoulder septic arthritis, where mortality rates ranged from 5% to 17% [41,52].

The current study's scope was confined to patients who underwent surgical drainage as part of their treatment for septic arthritis, with those receiving antibiotic therapy alone being excluded. This selective inclusion may introduce potential bias in the participant selection process. Furthermore, the analysis conducted in this study was limited to univariate analysis, focusing on the identification of individual prognostic factors contributing to complications in septic arthritis. A more comprehensive multivariate analysis would have allowed for the control and evaluation of multiple prognostic variables, thus reducing the impact of confounding factors. Regrettably, the sample size in this study did not provide sufficient data to perform such a comprehensive analysis. Nonetheless, it is important to highlight that this study represented a pioneering effort in assessing and pinpointing potential predictive factors linked to orthopedic and clinical complications in patients diagnosed with shoulder septic arthritis.

## Conclusions

The prevalence of positive findings associated with crystal arteriopathies appears to be decreasing when compared to research conducted in different geographical regions. This observation suggests that relying solely on the examination of synovial fluid under a microscope may not always yield positive results in the context of crystal arteriopathies. Therefore, it is advisable to explore and consider alternative diagnostic pathways and methods for the detection and evaluation of these conditions.
